# Anticoagulation quality through time in therapeutic range in Sub-Saharan Africa: a systematic review and meta-analysis

**DOI:** 10.3389/fmed.2025.1517162

**Published:** 2025-03-14

**Authors:** Desalegn Getnet Demsie, Zenaw Debasu Addisu, Chernet Tafere, Kebede Feyisa, Bereket Bahiru, Malede Berihun Yismaw, Getahun Mihret, Abere Tilahun, Desye Gebrie, Derbew Fikadu Berhe

**Affiliations:** ^1^Department of Pharmacy, College of Health Sciences, Bahir Dar University, Bahir Dar, Ethiopia; ^2^Department of Neurology, St. Peter Hospital, Addis Ababa, Ethiopia; ^3^Department of Anesthesia, College of Medicine and Health Sciences, Adigrat University, Adigrat, Ethiopia; ^4^Department of Pharmacy, College of Medicine and Health Sciences, Woldia University, Woldia, Ethiopia; ^5^Department of Pharmacology, Biomedical Division, and Center for Equity in Global Surgery, University of Global Health Equity, Kigali, Rwanda

**Keywords:** warfarin, Sub-Sahara Africa, anticoagulation, INR, TTR

## Abstract

**Background:**

The quality of anticoagulation with warfarin is often assessed through the time in therapeutic range (TTR). However, achieving optimal TTR and maintaining therapeutic INR levels presents significant challenges in Sub-Saharan Africa. This review aims to summarize the existing evidence on the quality of warfarin anticoagulation among patients in Sub-Saharan Africa.

**Method:**

We searched MEDLINE via Ovid, PubMed, Embase via Ovid, and Scopus, and citation analysis from Google Scholar. The review’s primary focus was therapeutic INR and TTR ≥ 65. Meta-analysis was conducted using R version 4.3.3. A mixed-effects meta-regression model was used to examine the influence of moderators, with heterogeneity estimated using *I*^2^ and prediction intervals (PI), and publication bias assessed through funnel plots and Egger’s test, with *p* < 0.05 indicating potential bias. The robustness of pooled proportions was tested using a leave-one-out sensitivity analysis. The preparation of this review adhered to the guidelines outlined in the PRISMA.

**Results:**

We identified 15 observational studies for inclusion in this systematic review and meta-analysis. Egger’s test confirmed an absence of publication bias across these studies. Sensitivity analyses showed consistency in individual therapeutic INR (pooled estimate: 0.37; range: 0.37–0.40) and TTR (pooled estimate: 0.16; range: 0.15–0.17), closely aligning with pooled proportions. Meta-analysis of high-quality TTR measurements yielded a pooled prevalence of 17% (*I*^2^ = 89%), with study-specific values ranging from 10 to 29% and predicted effect sizes between 0.05 and 0.34. The therapeutic INR was observed at a pooled prevalence of 40% (*I*^2^ = 86%; prediction interval: 0.16, 0.67).

**Conclusion:**

Warfarin therapy is associated with very low percentage of TTR suggests poor quality of anticoagulation management. Sensitivity analyses confirmed the robustness of these findings.

## Introduction

Warfarin, an oral anticoagulant, has been extensively used for decades to prevent and treat thromboembolic events such as stroke, deep vein thrombosis, and pulmonary embolism. While it offers significant clinical benefits, its management is challenging due to a narrow therapeutic window, which increases the risk of both major and minor bleeding complications (1, 2). Additionally, warfarin therapy is associated with effectiveness issues, including recurrent thromboembolic events and elevated mortality rates, particularly in patients who do not maintain therapeutic INR levels (3–6). Clinical outcomes related to warfarin therapy are closely tied to maintaining effective anticoagulation, which is often assessed through the calculation of Time in Therapeutic Range (TTR). TTR estimates the percentage of time a patient’s INR (International Normalized Ratio) remains within the target therapeutic range of 2–3. It serves as an essential tool for balancing the risks and benefits of warfarin use (7). However, consistently maintaining a therapeutic INR and achieving the recommended TTR (≥65%) can be challenging (8–10).

The uncertainty in maintaining consistent anticoagulation control requires patients to attend regular healthcare visits for INR monitoring and dose adjustments. Additionally, the warfarin dose needed to maintain a therapeutic INR can vary significantly between different patients and may fluctuate even within the same individual over time. Another complication is the potential for drug–drug interactions, which can affect the safety and effectiveness of warfarin by altering its metabolism (2, 11, 12). On top of this, genetic variations among patients influence how warfarin is metabolized, making it even more difficult to establish optimal dosing (13–15). Despite these complexities, warfarin remains the mainstay of anticoagulation therapy in Sub-Saharan Africa, primarily due to its affordability and availability (16, 17). It is particularly common in the treatment of patients with atrial fibrillation, valvular heart disease, or a history of thromboembolic events (18–22). Yet, in addition to warfarin’s pharmacodynamic and pharmacokinetic complexities, challenges specific to Sub-Saharan Africa—such as limited access to regular INR monitoring, insufficient healthcare infrastructure, and inconsistent patient adherence—further complicate the achievement and maintenance of therapeutic INR levels (20, 21, 23).

To the best of our knowledge, there is a notable lack of pooled data on critical anticoagulation parameters such as TTR and INR control in Sub-Saharan Africa. This evidence gap hinders efforts to optimize warfarin management and improve patient outcomes in the region. To address these challenges, this study undertakes a systematic review and meta-analysis of the available data on TTR and INR control in Sub-Saharan Africa. By synthesizing region-specific evidence, the study aims to generate actionable insights that can improve anticoagulation practices, reduce complications, and enhance patient safety. Ultimately, the findings could inform the development of tailored guidelines, leading to more effective management of patients receiving warfarin therapy across Sub-Saharan Africa.

## Methods

### Data sources and search strategy

A thorough systematic literature search was carried out using three databases: MEDLINE via Ovid, Embase via Ovid, and Scopus, and citation analysis from Google Scholar from the inception to July, 2024. Furthermore, we hand-searched of reference lists of the retrieved articles to locate any potential studies not captured in the database searches. The search strategy employed a comprehensive approach, utilizing both Medical Subject Heading (MeSH) terms and a range of relevant keywords for warfarin, and anticoagulation control (Supplementary Table 1). This systematic review and meta-analysis was not registered in any registration databases.

### Eligibility criteria

The inclusion criteria for this study were designed to encompass a comprehensive range of observational studies (case–control, clinical trial, prospective, and retrospective). However, the absence of case–control and clinical trials during our search period limited the study design to a few prospective and retrospective studies. Furthermore, the criteria included studies that reported the proportion of patients with therapeutic, subtherapeutic, and supratherapeutic levels of INR values, along with measurements of TTR. Exclusive consideration was accorded to articles published in English.

In a deliberate exclusionary approach, patients with mechanical or prosthetic heart valve, and studies delving into the effects of anticoagulation arising from the concomitant use of warfarin and other medications were excluded. Moreover, a refined focus on the scope of safety-related outcomes led to the exclusion of reports addressing aspects beyond anticoagulation control. This exclusion encompassed pharmacogenomic analyses, qualitative studies, review articles, and unpublished works.

### Process for screening studies

Two researchers (T.C and A.Z.D) independently screened articles for compliance with eligibility criteria among the retrieved studies. The initial selection was based on the title and abstract. Subsequently, especially when the title and abstract provided insufficient information, the full text of studies was reviewed to assess the relevance of the papers. In instances of disagreements, the other author (BB) facilitated a discussion to resolve conflicting ideas. If a consensus could not be reached, additional independent adjudication involving all authors was considered.

### Data extraction

Data extraction was conducted employing a standardized data entry form by two researchers (TC and D.G.D). The collected information encompassed fundamental sociodemographic and clinical characteristics of patients, such as author and publication year, study design, follow-up period, sample size, common indication(s) for anticoagulation, and prevalent comorbidities. In addition to these characteristics, a thorough examination of the anticoagulation profile was carried out for patients undergoing warfarin therapy. This included the measurement of INR values and assessments of TTR.

### Quality assessment of studies

The Newcastle-Ottawa-Scale (NOS) for cross sectional and cohort studies was employed to assess the quality of observational studies. Using NOS, we assessed whether the selection of participants, comparability, and outcome of the research were designed as per the critical appraisal tool. The tool has three domains with a total score of nine; the selection domain was given a maximum of four stars, comparability two scores and outcome measure three stars. Each parameter under the domains was scored a maximum of one star. Studies with quality score of 7–9, 5–6, and 0–4 was rated as high, moderate and low, respectively (24).

### Outcome measurement

The primary outcome was anticoagulation quality expressed as percentage of time the patient’s INR was within target values (i.e., TTR) and INR value with the indicated therapeutic value. If TTR < 65%, the therapeutic efficacy of warfarin cannot be achieved, and the quality of anticoagulation is poor (25). INR value beyond the standard 2.0–3.0 is also considered poor quality anticoagulation (25, 26).

### Meta regression

This mixed-effects meta-regression model assessed the impact of publication year, sample size, setting (cardiac center vs. medical clinic), and follow-up period on INR and TTR in patients using warfarin. The influence of moderators—including publication year, follow-up period, sample size, and study setting—was analyzed to determine their potential effect on anticoagulation control.

### Sensitivity analysis

We conducted a sensitivity analysis on clinical outcomes using a leave-one-out technique to evaluate each study’s impact on the overall pooled estimate. The sensitivity analysis report examines the robustness of the meta-analysis findings by sequentially omitting each study to observe the impact on the overall proportion estimate. First, we developed a meta-analysis model with the inverse variance method and Freeman-Tukey transformation to assess the pooled outcomes. We then applied the **metainf** function to systematically omit each study individually, generating a forest plot to observe any significant shifts in the results.

### Heterogeneity and publication bias

Prediction intervals (PIs) were employed in meta-analysis to estimate the range within which the true effect sizes of future studies are likely to fall, incorporating both the uncertainty in the pooled effect estimate and the variability among study results. For interpretation, a wide prediction interval suggests high heterogeneity, indicating that future studies may report a broad range of effect sizes, while a narrow interval reflects greater consistency across studies. Additionally, *I*^2^ values were used to quantify the level of heterogeneity, although they provide less reliable evidence compared to prediction intervals. Heterogeneity was determined as low, moderate, and high using I-square (*I*^2^) values of *I*^2^ < 25, 25% < *I*^2^ < 50%, *I*^2^ > 50%, respectively. We evaluated publication bias using funnel plots and Egger’s test, considering a *p*-value below 0.05 as indicative of publication bias. Publication bias was assessed using funnel plots and Egger’s test, with statistical significance defined as *p* < 0.05.

### Statistical analysis

All analyses were carried out using R version 4.3.3. A meta-analysis of the pooled proportions was performed using the *metaprop()* function from the *meta* package. This method was used to calculate the proportion of patients with good TTR and therapeutic INR across studies. The data input consisted of the number of Events and the total number of participants in each study. The inverse-variance method (method = “inverse”) was applied to weight the studies based on their sample size and variance. The *Freeman-Tukey transformation (PFT)* was used to stabilize the variance and improve the accuracy of the pooled estimates, especially for proportions. The random-effects model was chosen to account for between-study heterogeneity, and the results were summarized as an overall pooled estimate of the proportion of patients achieving good TTR or therapeutic INR. This method also provided the prediction interval, which reflects the expected range of treatment effects in future studies, taking into account the observed between-study variability.

To visualize the results, a forest plot was generated using the forest () function from the meta package. The studies were represented by red squares, with the size of each square proportional to the weight of each study. The confidence intervals for each study were depicted as horizontal lines extending from the squares. The overall pooled estimate was represented by a diamond shape, and the prediction interval was included to show the expected range of future effects. This forest plot was created with both fixed-effects and random-effects models to compare the overall estimates while accounting for between-study heterogeneity.

A sensitivity analysis was conducted using the *metainf()* function from the *meta* package. A meta-regression analysis was performed using the *metafor* package. Moderator variables such as publication year, sample size, setting, and follow-up duration were included in the meta-regression model to determine their impact on the outcome. The meta-regression model was built using the *rma* () function, with effect sizes as the dependent variable and the moderators as independent variables.

To assess the possibility of publication bias, Egger’s test was conducted using the *metabias*() function from the meta package. Additionally, a funnel plot was created using the *funnel*() function to visually inspect the distribution of study estimates.

## Results

### Study identification and selection

**Figure 1:**
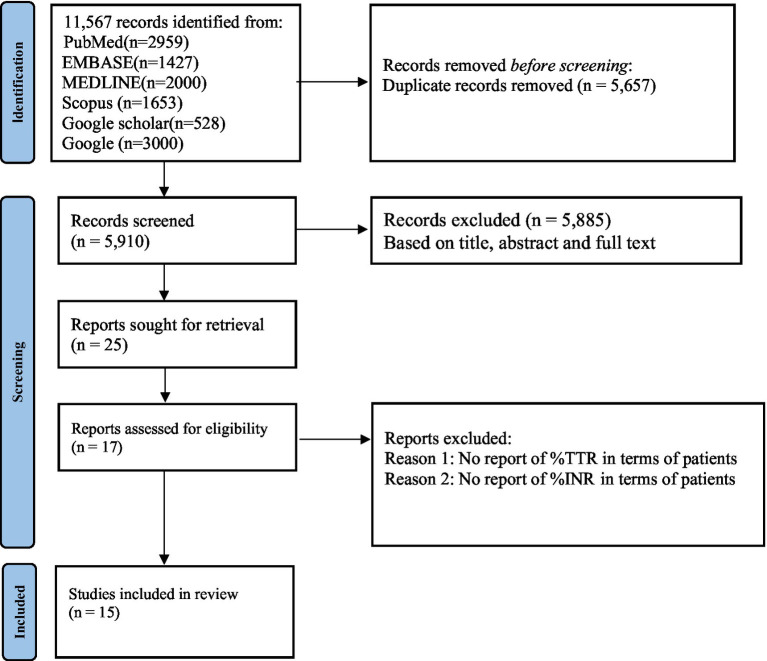
Preferred reporting items for systematic reviews and meta-analyses (PRISMA).

Figure 1 presents the PRISMA flow diagram outlining the study selection process for this systematic review and meta-analysis. The initial search identified a total of 11,567 records. After removing duplicates, 5,910 records remained. These records underwent title, abstract and full-text screening, from which 5,885 were excluded based on irrelevance, leaving 25 full-text articles for further eligibility assessment. During the full-text review, 10 studies were excluded due to reasons such as insufficient outcome data or lack of adherence to inclusion criteria, resulting in a final selection of 15 studies that met the criteria for inclusion in the quantitative synthesis.

### Study characteristics

Most of the included studies were from Ethiopia (*n* = 5) (19, 23, 27–29), South Africa (*n* = 4) (3, 22, 30, 31), and Botswana (*n* = 2) (32, 33) (Table 1). The studies involved adult patients (≥18 years). The follow-up durations differed significantly, with some studies having a short-term follow-up of 3–6 months (19, 22), while others had longer follow-up periods extending to 2 or even 5 years (3, 34). The mean and median ages of study populations ranged from mid-30s to mid-60s, with most studies reporting a mean age between 35 and 64 years.

**Table 1:** Demographic and methodological characteristics of included studies.

Author and year	Country	Study design	Study population	Follow-up time	Mean or median age	Setting	Sample size	Common indications
Getachew et al. (19)	Ethiopia	Retrospective	Adult patients (≥18 years)	3 months and above	Mean: 57	Private cardiac centers	374	AF, CRHF
Fenta et al. (23)	Ethiopia	Retrospective	Adults outpatients	12 months	Mean: 35.3	Cardiac and hematology Clinics	360	VHD, AF, MVR
Anakwue et al. (71)	Nigeria	Retrospective	Adult patients	5 years	Mean: 53.4	Teaching Hospital	26	DVT/PE, CHF with AF mitral valve disease with AF
Sonuga et al. (22)	South Africa	Retrospective	Adult patients	6 months	Mean: 62 (for male), Median: 66 (for female)	INR Clinic	136	DVT, AF. VHD, mHVR, DVT, PE, hyper coagulation, atrial flutter, Cardiomyopathy/LV thrombosis
Yimer et al. (29)	Ethiopia	Retrospective	Adult outpatients	2 years	Mean: 56.4	Anticoagulation Clinic	300	AF
Mwita et al. (22)	Botswana	Retrospective	Adults	2 years	Median: 46 (IQR, 35–58) years	General medical clinics	410	MHV, DVT, AF, ICT, PH
Liyew et al. (27)	Ethiopia	Retrospective	Adults	≥6 months	Mean: 18	Cardiology and Hematology Clinics	338	AF, VTE, PHV, ICT
Prinsloo et al. (31)	South Africa	Retrospective	Adult patients	12 months	Median: 56	INR Clinic	191	AF/AFib, VTE, MPV
Botsile and Mwita (32)	Botswana	Retrospective	Patients aged ≥18 years	5 months	Mean: 42	INR Clinic	142	MHV
Mariita et al. (60)	Kenya	Retrospective	Adults	<3 months 3 months – 1 year >1 year	Median: 56	Medical	147	VTE
Ahmed et al. (36)	Sudan	Retrospective	Adult patients	12 months	Mean: 41.8	Anticoagulation Clinic	135	MVR, AVR, DVR
Masresha et al. (28)	Ethiopia	Retrospective	Adult patients	2 years	Mean: 44.33	Outpatient department	202	AF, VHD, DVT, PE
Ouali et al. (21)	Tunisia	Prospective	≥18 years	1 year	Mean: 64.3	Cardiac clinic	915	AF
Jonkman et al. (20)	Namibia	Retrospective	Adults	1 year	NA	Medical clinic	215	DVT, PE, AF, CVA, AVR, LVT, MVR, DVR
Ebrahim et al. (3)	South Africa	Retrospective	Adult out patients	6 years	Median: 55 (IQR 44–64)	Warfarin anticoagulation Clinic	363	AF, VHD, PE, VTE

DVT/PE, deep vein thrombosis/pulmonary embolism; CHF with AF, congestive heart failure with atrial fibrillation; mitral valve disease with AF; VHD, valvular heart disease; mHVR, mitral heart valve replacement; LV, left ventricle; MHV, mitral heart valve; ICT, intracardiac thrombus; PH, pulmonary hypertension; VTE, venous thromboembolism; PHV, prosthetic heart valve; AFib, atrial fibrillation; MPV, mitral prosthetic valve; nvAF, non-valvular atrial fibrillation; PHV, prosthetic heart valve; vAF, valvular atrial fibrillation; HF, heart failure; MHV, mitral heart valve; MVR, mitral valve replacement; AVR, aortic valve replacement; DVR, double valve replacement.

Regarding sample sizes, the studies varied widely, from as small as 26 patients in Anakwue et al. (34) to as large as 915 patients in the study by Ouali et al. (21). The majority of the studies had sample sizes ranging from 100 to 400 participants. The common indications for warfarin use include deep vein thrombosis (DVT), atrial fibrillation, mechanical valve replacement, venous thromboembolism, and pulmonary embolism (Table 1).

### Anticoagulation control

As indicated in Table 2, for INR values, the percentage of patients within the therapeutic range (INR 2–3) varied from 29.0% in Fenta et al. (23) to 51.5% in Ahmed et al. (35), with other studies, such as Liyew et al. (27) and Mariita et al. (36), reporting values of 33 and 43.5%, respectively. The proportion of patients with subtherapeutic INR (<2.0) ranged from 18.8% in Fenta et al. (23) to 49% in Liyew et al. (27). Supratherapeutic INR (> 3) was less common but still present in varying degrees, with values ranging from 10.3% in Sonuga et al. (22) to 52.2% in Fenta et al. (23).

**Table 2:** Anticoagulation metrics and outcomes in warfarin-treated patients.

First author	INR values			TTR	
	Therapeutic (2–3)	Subtherapeutic (<2.0)	Supratherapeutic (>3)	Good TTR	Poor TTR
Anakwue et al. (71)	38	NA	NA	NA	NA
Sonuga et al. (22)	48.5	41.2	10.3	NA	NA
Liyew et al. (27)	33	49	18	13	87
Ahmed et al. (36)	51.5	NA	NA	NA	NA
Mariita et al. (60)	43.5	39.5	17	NA	NA
Getachew et al. (19)	NA	NA	NA	25.67	74.33
Fenta et al. (23)	29.0	18.8	52.2	NA	NA
Yimer et al. (29)	NA	NA	NA	12.67	87.3
Ebrahim et al. (3)	NA	NA	NA	25.1	74.9
Jonkman et al. (20)	NA	NA	NA	10	90
Masresha et al. (28)	NA	NA	NA	29.2	70.8
Ouali et al. (21)	NA	NA	NA	12.02	87.98
Botsile and Mwita (32)	NA	NA	NA	14.8	85.2
Mwita et al. (22)	NA	NA	NA	14.9	85.1
Prinsloo et al. (31)	NA	NA	NA	17.80%	82.2

The percentage of patients with good TTR was generally low across the studies. The highest proportion of patients with good TTR was reported by Jonkman et al. (20), at 29.2%. Other studies, including Yimer et al. (29) and Ouali et al. (21), reported good TTR in 12.67 and 12.02% of patients, respectively. In contrast, poor TTR was much more common, with values ranging from 70.8% in Masresha et al. (28) to 87.98% in Ouali et al. (21) (Table 2).

### Meta analysis of anticoagulation control

#### Quality assessment and publication bias

**Figure 2:**
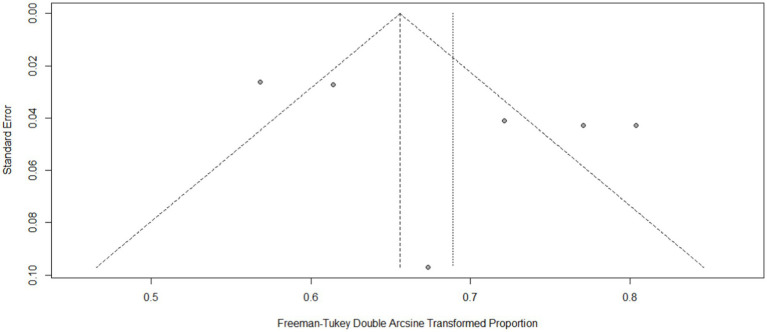
Funnel plot of therapeutic INR.

**Figure 3:**
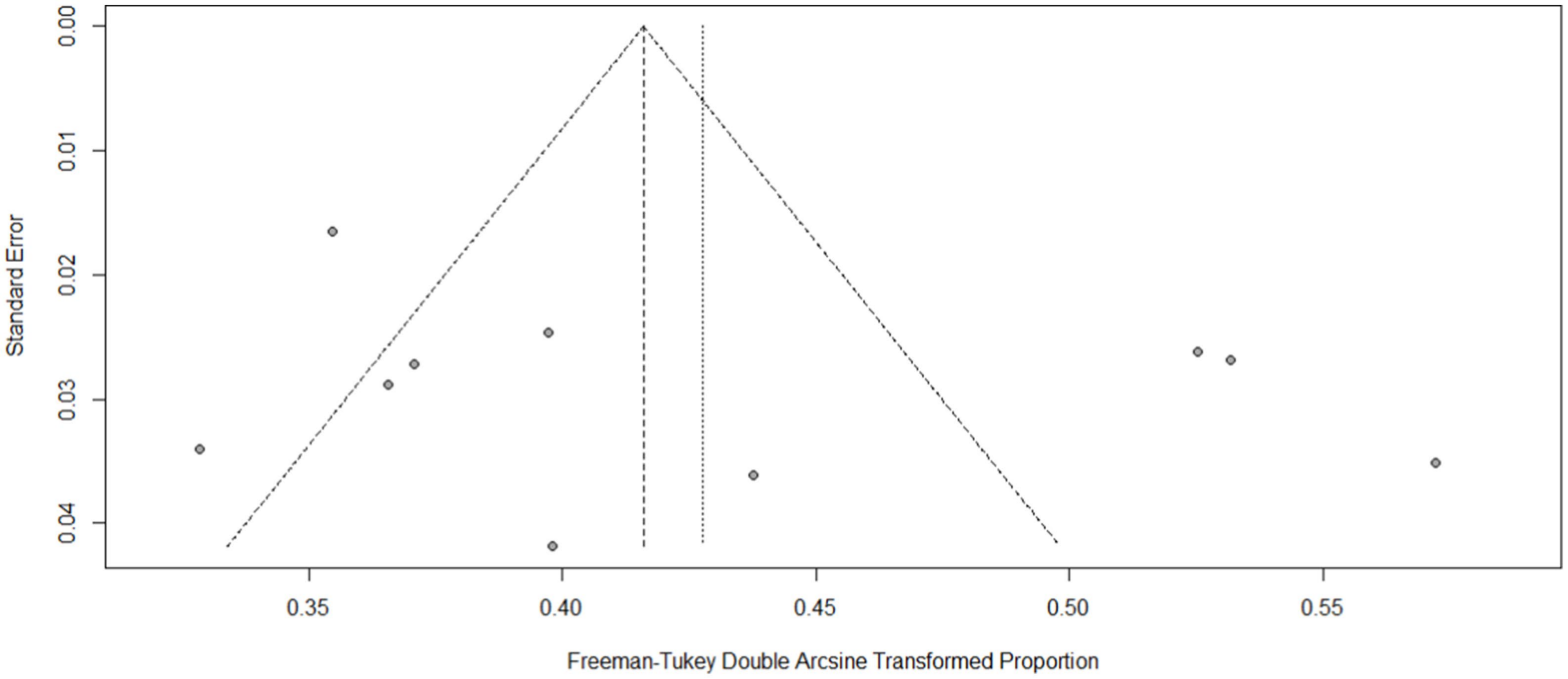
Funnel plot of good-TTR.

Funnel plots and Egger’s test yielded statistically insignificant results for INR (Egger’s test: *p* = 0.1935) (Figure 2), and TTR (Egger’s test: *p* = 0.3220) (Figure 3). All of the observational studies scored from 5 to 8 on the Newcastle–Ottawa Scale criteria and were included in the quantitative analysis. Eight observational studies were considered to be of high quality (Newcastle–Ottawa score ≥ 7; Supplementary Table 2).

#### Meta-regression

For therapeutic INR, the mixed-effects meta-regression explained significant residual heterogeneity (QE = 0.4, *p* < 0.52), but the combined test for moderators was statistically significant (QM = 34, *p* = 0.0001) (Supplementary material). Publication year (estimate = 0.0478, *p* = 0.0365) and follow-up duration (estimate = 0.0494, *p* = 0.0160) were associated with heterogeneity in TTR outcomes, while sample size was associated with heterogeneity in therapeutic INR (*p* = 0.0023) (Table 3).

**Table 3:** Meta-regression analysis of moderator effects on clinical outcomes in warfarin therapy.

Anticoagulation control	Moderators	Estimate	SE	*p*-value
Good TTR	Publication year	0.0478	0.0229	0.0365
	Sample size	−0.0001	0.0001	0.3187
	Setting	−0.0367	0.0565	0.5162
	Follow-up	0.0494	0.0205	0.0160
	Publication year	0.0060	0.0091	0.5091
Therapeutic INR	Sample size	−0.0007	0.0002	0.0023
	Setting	0.0698	0.0481	0.1464
	Follow-up	−0.0212	0.0228	0.3531

SE, standard error; TTR, time in therapeutic range; INR, international normalized ratio.

### Sensitivity analysis

**Figure 4:**
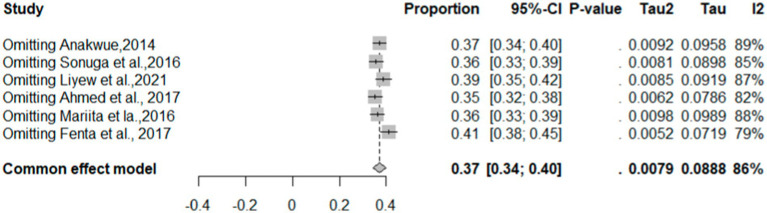
Sensitivity analysis of therapeutic INR.

**Figure 5:**
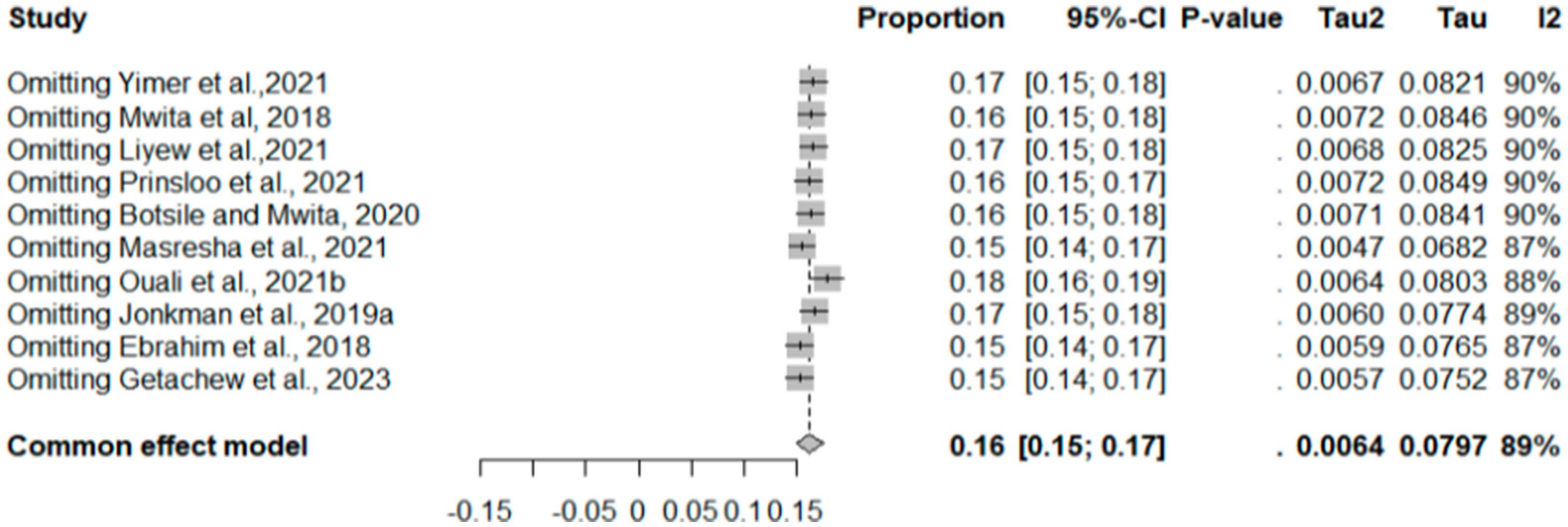
Sensitivity analysis of good TTR.

The common-effect model indicates a pooled therapeutic INR proportion estimate of 0.37 (95% CI: 0.37–0.40) (Figure 4). Sensitivity analysis shows that with each omitted study, the proportion varies from 0.35 to 0.41, with *I*^2^ values ranging between 79 and 89%. Notably, omitting “Fenta et al. (23)” reduces the *I*^2^ to 79%. The pooled proportion of good TTR was 0.16 (*I*^2^: 89), with which individual proportions varies from 0.15 to 0.17 (*I*^2^: 87–90) (Figure 5).

### Pooled proportion of therapeutic INR, and good TTR

**Figure 6:**
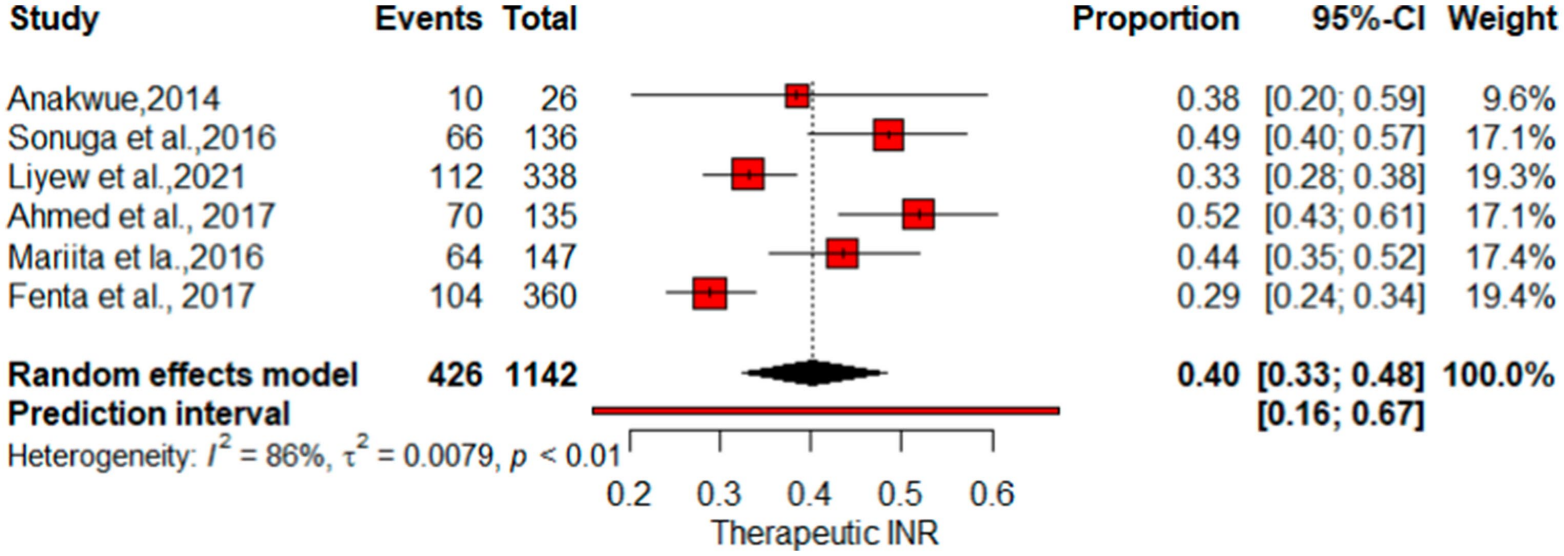
Forest plot of therapeutic INR.

**Figure 7:**
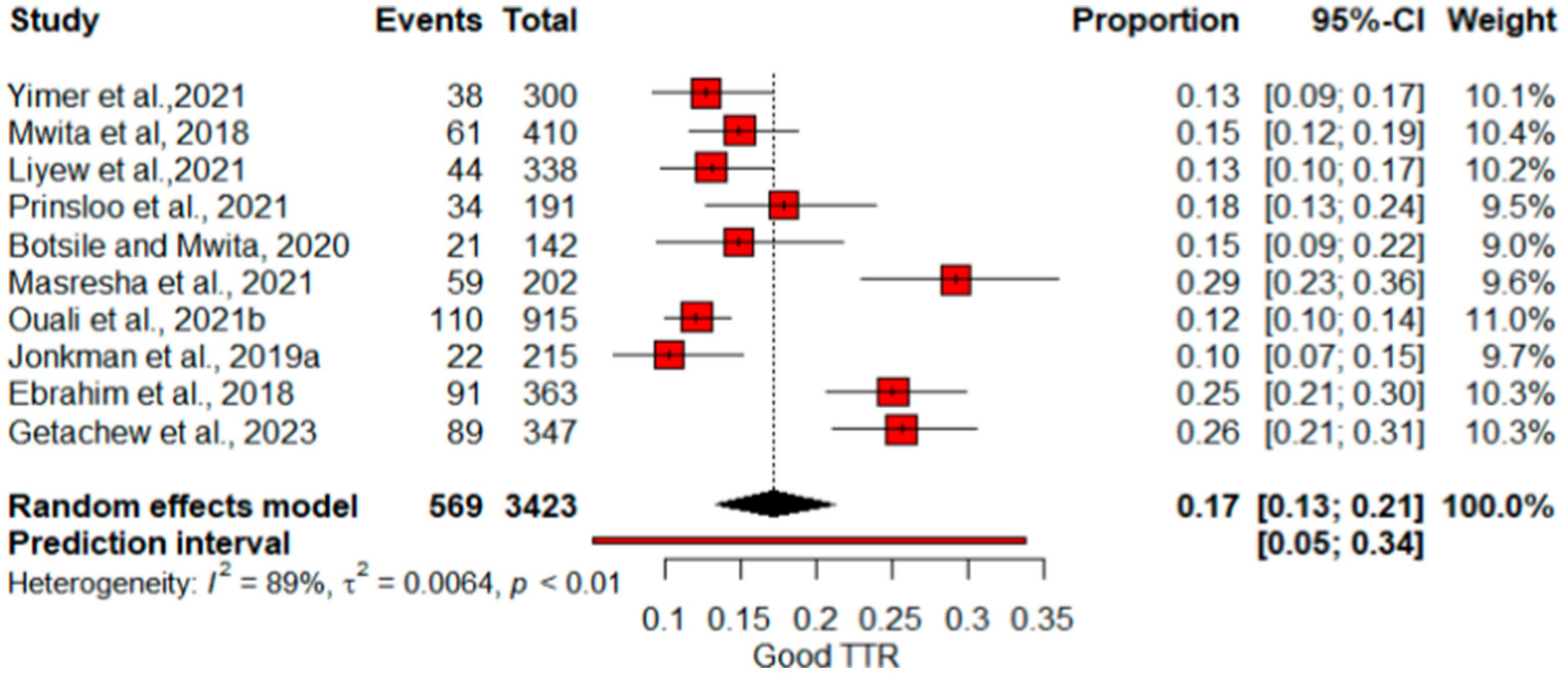
Forest plot of Good TTR.

As indicated in Figure 6, the pooled therapeutic-INR measure of patients on warfarin therapy was 0.40 (40%) (95% CI: 0.33, 0.48; *I*^2^ = 86%). PI for effect sizes spanned from 0.16 to 0.67. The pooled proportion of patients demonstrating good-quality TTR measurements was 0.17 (95%CI: 0.13–0.21). The proportion of Good TTR values range from 0.1 to 0.29, with effect size predicted from 0.05 to 0.34 (Figure 7).

## Discussion

This systematic review and meta-analysis, encompassing data from 15 observational studies, sought to comprehensively evaluate therapeutic INR and good TTR associated with warfarin anticoagulation. The proportions of patients with INR values and TTR were employed to gauge the quality of anticoagulation. The overall percentage of patients achieving therapeutic INR levels was 40% (*I*^2^ = 86%), suggesting that the remaining 60% had either supratherapeutic or subtherapeutic levels. Sensitivity analysis produced a pooled proportion estimate of 0.37 (95% CI: 0.37, 0.40). Exclusion of individual studies yielded similar proportions, ranging from 0.35 to 0.41, indicating that no single study significantly influenced the overall estimate. However, heterogeneity indicators, particularly *I*^2^, remained high at 79–89% across most omissions, underscoring substantial variation among studies—likely due to differences in study populations, methodologies, or other factors rather than random variation. Notably, exclusion of “Fenta et al., (23)” lowered *I*^2^ to 79%, with the highest *I*^2^ (89%) observed after omitting “Anakwue et al. (34).” Meta-regression analysis identified publication year (estimate = 0.0478, *p* = 0.0365) and follow-up duration (estimate = 0.0494, *p* = 0.0160) as contributors to heterogeneity in TTR outcomes.

The pooled percentage of therapeutic INR showed variations compared with other studies, where the estimate was lower than the reported mean percent within the therapeutic range for INR (67%) (37) and a study by Singer et al. at 55.2% (38). Nevertheless, it surpasses the results of the multicenter international prospective analysis of the Global Anticoagulant Registry in the FIELD–Atrial Fibrillation with an East and Southeast Asia analysis, which indicated that 31.1% of patients had therapeutic INR values (39). For optimal anticoagulation safety and efficacy of warfarin, INR values need to be within the therapeutic range for at least 65% of the time, suggesting that despite regular INR checkups, patients may not achieve good INR control and effectiveness (3). Figure 7 illustrates that the proportion of patients achieving a high-quality TTR was 17%, underscoring the unpredictable impact of warfarin in certain users.

Considerable disparity in proportions of patients with low anticoagulation quality underscores the importance of considering regional and contextual factors, as well as variations in study populations and methodologies (40–42). Findings indicated that the use of warfarin does not guarantee the maintenance of target INR values, leading to poor TTR records (43, 44). Presumably, the unpredictable pharmacokinetics and dynamics of warfarin, coupled with its narrow therapeutic index, expose patients to the risk of bleeding and thromboembolic complications (6). This makes warfarin a leading cause of adverse drug reaction-related medical admissions in countries in the Sub-Saharan region such as South Africa (45). The diverse range of reported rates highlights the need for further exploration into factors influencing anticoagulation quality and treatment outcomes (29, 42, 46). In this context consideration of patients’ age, and weight, could decrease risk of suboptimal anticoagulation (8). Warfarin therapy’s toxicity or reduced effectiveness is often linked to genetic factors, particularly polymorphisms in cytochrome P450 isozymes, such as CYP2C9 (9, 10), VKORC1 (10), and CYP4F2 (10) causes inter-individual variability in warfarin metabolism. CYP2C9*2 and CYP2C9*3 variants significantly influence warfarin metabolism and subsequently the required dose of warfarin. Patients carrying these variants required 39% lower warfarin maintenance dose (9), yet the limited application of pharmacogenomics in African clinical settings emphasizes the need for incorporating pharmacogenetic profiling to ensure the effective and safe administration of therapeutics (14).

The COVID-19 pandemic further disrupted anticoagulation management, restricting patient access to routine medical services (47–49). Most of the studies included in this meta-analysis were conducted during periods of high COVID-19 burden (Figure 7), when lockdowns, movement restrictions, and fear of infection led to decreased hospital visits and poor INR control. Medication adherence declined, and prescription refills were delayed, exacerbating anticoagulation instability (50). In response, the UK’s National Institute for Health and Care Excellence (NICE) recommended switching patients from warfarin to DOACs to reduce the need for frequent monitoring during the pandemic (47). The use of direct oral anticoagulants (DOACs) offers a viable alternative to warfarin, with advantages including a more predictable anticoagulation effect, fewer drug–drug interactions, and no need for frequent INR monitoring (51–53). A meta-analysis has shown that DOACs, such as rivaroxaban, dabigatran, and apixaban, are associated with a significantly lower risk of intracranial hemorrhage compared to Vitamin K antagonists (VKAs) (54–56). However, in Sub-Saharan Africa, the adoption of DOACs is hindered by economic barriers, limited awareness among healthcare providers, and inadequate insurance coverage for newer anticoagulants (44, 57–59). Additionally, the limited availability of reversal agents raises concerns about their safety in emergency situations (52).

Moreover, poor patient adherence to anticoagulation therapy, driven by financial constraints, long travel distances, and work commitments, significantly impacts INR control (60–63). Systemic healthcare limitations—including the absence of specialized anticoagulation clinics, inadequate INR monitoring infrastructure, and a shortage of trained personnel—further exacerbate these challenges (60, 61).

Healthcare systems efforts should focus on implementing close monitoring (64). Achieving the first therapeutic INR—specifically reaching an INR ≥ 1.8 within 6 days of starting oral warfarin—through close monitoring and dose adjustments may shorten hospital stays and enhance patient safety (65). This aligns with findings indicating that adjusted-dose warfarin therapy in patients with AF reduces stroke risk by nearly 60% (66). Nevertheless, stringent monitoring may be affected by income disparities, which also play a crucial role in shaping anticoagulation management strategies (67). Dedicated anticoagulation clinics in high-income countries have demonstrated improvements in INR control, yet their scalability in low- and middle-income countries (LMICs) remains uncertain (63). Pharmacist-Managed Anticoagulation Clinics (PMACs) have shown promise in optimizing INR control (23). Additionally, expanding access to point-of-care INR testing and implementing standardized warfarin dosing protocols could enhance anticoagulation outcomes while alleviating burdens on both patients and healthcare providers (67–69). Findings from high-income and urban settings may not directly apply to rural areas, where specialized clinics are scarce, and distinct barriers exist. Rural patients often contend with poor transportation, underdeveloped infrastructure, and reliance on mobile clinics with intermittent services. Healthcare facilities in these regions are frequently staffed by community service officers and nurses with limited supervision and high turnover. INR testing is further complicated by distant laboratories, delayed results, and poor telecommunication access for dose adjustments. Given that most South Africans depend on under-resourced primary healthcare centers, these challenges are particularly significant (31).

The development and implementation of dosing protocols for initiation and maintenance/adjustment that consider locally relevant factors, as emphasized by Parbhoo and Jacobson in 2019 may be play a role in anticoagulation control. Additionally, raising public awareness, and addressing language barriers collectively contribute to enhancing patient safety, as highlighted by Stambler and Ngunga (70). Monitoring drug–drug interactions and accounting for the impact of concomitant disease conditions are crucial considerations in achieving optimal anticoagulation control. An Ethiopian study suggests that coadministration of warfarin with 1 or 2 drugs may result in diminished TTR measures. Furthermore, Yimer et al. (29) reported that heart failure contributes to suboptimal anticoagulation control.

In conclusion, our systematic review and meta-analysis shed light on the quality of anticoagulation with warfarin in Sub-Saharan Africa. The findings revealed a concerning low anticoagulation control among the studied population. A notable proportion of patients exhibited suboptimal therapeutic INR values and low TTR, indicating challenges in maintaining adequate anticoagulation control. These results underscore the need for improved monitoring and management strategies in this region. Further research and healthcare interventions are warranted to enhance the safety and efficacy of warfarin therapy, ensuring better outcomes for patients requiring anticoagulation in Sub-Saharan Africa. Improving anticoagulation control in resource-limited settings requires a multifaceted approach, including the decentralization of anticoagulation care, increased availability of anticoagulants, and strengthened support for both healthcare providers and patients.

### Strength and limitation of the study

This SRMA highlights the prevalence of therapeutic INR and TTR in SSA and applies rigorous methods to evaluate the robustness of findings, investigate sources of heterogeneity, and identify key predictors of variation, such as PIs. The study provides valuable insights into the burden of poor anticoagulation control across SSA, offering data to inform healthcare strategies. A significant limitation of this SRMA is its reliance on a limited number of prospective studies, alongside many retrospective studies, with a notable absence of clinical trials or case–control studies. Additionally, small sample sizes in included studies introduce the risk of selection bias, potentially impacting the representativeness and generalizability of the findings. The inability to assess temporal trends or adjust for confounding variables also restricts the depth of the analysis. Sparse data availability and cultural variability across SSA pose further challenges to consistency and interpretability. To address these limitations, future research should prioritize larger, longitudinal studies and collaborative efforts to enhance the robustness and applicability of findings in SSA’s public health context.

## Data Availability

The original contributions presented in the study are included in the article/Supplementary material, further inquiries can be directed to the corresponding author.
