# Supplementary material for: Anticoagulation quality through time in therapeutic range in Sub-Saharan Africa: a systematic review and meta-analysis
Source: Front Med (Lausanne). 2025 Mar 14;12:1517162. doi: 10.3389/fmed.2025.1517162 (PMC11949880; doi:10.3389/fmed.2025.1517162)
Supplement: Supplementary file 1 [file Table_1.docx]

|  | PubMed |  |
| --- | --- | --- |
| #1 | "venous thrombosis"[Mesh] OR "pulmonary embolism"[Mesh] OR "venous thromboembolism"[Mesh] OR "ischemic stroke"[Mesh] OR "myocardial infarction"[Mesh] OR "treatment outcome"[Mesh] OR "international normalized ratio [MeSH" OR safety [Mesh] OR hemorrhage [Mesh] OR mortality [Mesh] OR ("Deep venous thrombosis" OR DVT OR "pulmonary embolism" OR PE OR "Ischemic stroke" OR "venous thromboembolism" OR VTE OR "myocardial infarction" OR MI OR "treatment outcome" OR TTR OR INR OR  "International Normalized Ratio" OR "Time in Therapeutic Range" OR  "Clinical effectiveness" OR efficacy OR safety OR bleeding* OR Hemorrhage* OR "Major bleeding" OR "intracranial hemorrhage" OR "gastrointestinal bleeding" OR mortality OR hospital* OR "intracranial bleeding") tiab | 13,715,900 |
| #2 | Anticoagulants [Mesh] OR warfarin[Mesh] OR (Anticoagulant OR "anticoagulant drugs" OR warfarin OR Coumarin OR Coumadin OR "vitamin K antagonist" OR  VKA OR "oral anticoagulant" OR OAC) tiab | 351,667 |
| #3 | Africa central OR Africa eastern OR Africa southern OR Africa western OR Africa northern OR low-income country OR developing country OR middle-income country OR Sub-Sahara OR Angola OR Benin OR Botswana OR Burkina Faso OR Burundi OR Cameroon OR Cape Verde OR Central African Republic OR Chad OR Comoros OR Congo OR Brazzaville OR Cote d'Ivoire OR Djibouti OR Equatorial Guinea OR Eritrea OR Ethiopia OR Gabon OR Gambia OR Ghana OR Guinea OR Guinea-Bissau OR Kenya OR Lesotho OR Liberia OR Madagascar OR Malawi OR Mali OR Mauritania OR Mauritius OR Mozambique OR Namibia OR Niger OR Nigeria OR Rwanda OR Sao Tome and Principe OR Senegal OR Seychelles OR Sierra Leone OR Somalia OR South Africa OR Sudan OR Swaziland OR Tanzania OR Togo OR Uganda OR Western Sahara OR Zambia OR Zimbabwe | 894,235 |
| #4 | #1AND#2AND#3 | 2,959 |

Supplemental Table 1: Search strategy for PubMed

**Table 1**: Search strategy for MEDLINE (cont’d)

|  | Ovid MEDLINE |  |
| --- | --- | --- |
| #1 | exp "venous thrombosis"/ OR exp "pulmonary embolism"/ OR exp "venous thromboembolism"/ OR exp "ischemic stroke"/ OR exp "myocardial infarction"/ OR exp "treatment outcome"/ OR "international normalized ratio[MeSH" OR exp safety/ OR exp hemorrhage/ OR exp mortality/ OR ("Deep venous thrombosis" OR DVT OR "pulmonary embolism" OR PE OR "ischemic stroke" OR "venous thromboembolism" OR VTE OR "myocardial infarction" OR  MI OR "treatment outcome" OR TTR OR INR OR "International Normalized Ratio" OR "Time in Therapeutic Range" OR "clinical effectiveness" OR efficacy  OR safety OR bleeding* OR Hemorrhage* OR "major bleeding" OR "intracranial hemorrhage" OR "gastrointestinal bleeding" OR mortality OR hospital* OR  "Intracranial bleeding”) tiab |  |
| #2 | exp Anticoagulants/ OR exp warfarin/ OR (Anticoagulant OR "anticoagulant drugs" OR warfarin OR Coumarin OR Coumadin OR "vitamin K antagonist" OR  VKA OR "oral anticoagulant" OR OAC) tiab |  |
| #3 | Africa central OR Africa eastern OR Africa southern OR Africa western OR Africa northern OR low-income country OR developing country OR middle-income country OR Sub-Sahara OR Angola OR Benin OR Botswana OR Burkina Faso OR Burundi OR Cameroon OR Cape Verde OR Central African Republic OR Chad OR Comoros OR Congo OR Brazzaville OR Cote d'Ivoire OR Djibouti OR Equatorial Guinea OR Eritrea OR Ethiopia OR Gabon OR Gambia OR Ghana OR Guinea OR Guinea-Bissau OR Kenya OR Lesotho OR Liberia OR Madagascar OR Malawi OR Mali OR Mauritania OR Mauritius OR Mozambique OR Namibia OR Niger OR Nigeria OR Rwanda OR Sao Tome and Principe OR Senegal OR Seychelles OR Sierra Leone OR Somalia OR South Africa OR Sudan OR Swaziland OR Tanzania OR Togo OR Uganda OR Western Sahara OR Zambia OR Zimbabwe |  |
| #4 | #1AND#2AND#3 |  |

**Table 1**: Search strategy for EMBASE (cont’d)

|  | **EMBASE** |  |
| --- | --- | --- |
| #1 | exp "venous thrombosis"/ OR exp "pulmonary embolism"/ OR exp "venous thromboembolism"/ OR exp "ischemic stroke"/ OR exp "myocardial infarction"/ OR exp "treatment outcome"/  OR exp safety/ OR exp hemorrhage/ OR exp mortality/ OR ("Deep venous thrombosis" OR DVT OR "pulmonary embolism" OR PE OR "ischemic stroke" OR "venous thromboembolism" OR VTE OR "myocardial infarction" OR MI OR "treatment outcome"   OR TTR OR INR OR "International Normalized Ratio" OR "Time in Therapeutic Range"   OR "clinical effectiveness" OR efficacy OR safety OR bleeding* OR Hemorrhage* OR "major bleeding" OR "intracranial hemorrhage" OR "gastrointestinal bleeding"  OR mortality OR hospital* OR "intracranial bleeding")tiab |  |
| #2 | exp Anticoagulants/ OR exp warfarin/ OR (Anticoagulant OR "anticoagulant drugs" OR warfarin OR  Coumarin OR Coumadin OR "vitamin K antagonist" OR VKA OR "oral anticoagulant" OR OAC) tiab |  |
| #3 | Africa central OR Africa eastern OR Africa southern OR Africa western OR Africa northern OR low-income country OR developing country OR middle-income country OR Sub-Sahara OR Angola OR Benin OR Botswana OR Burkina Faso OR Burundi OR Cameroon OR Cape Verde OR Central African Republic OR Chad OR Comoros OR Congo OR Brazzaville OR Cote d'Ivoire OR Djibouti OR Equatorial Guinea OR Eritrea OR Ethiopia OR Gabon OR Gambia OR Ghana OR Guinea OR Guinea-Bissau OR Kenya OR Lesotho OR Liberia OR Madagascar OR Malawi OR Mali OR Mauritania OR Mauritius OR Mozambique OR Namibia OR Niger OR Nigeria OR Rwanda OR Sao Tome and Principe OR Senegal OR Seychelles OR Sierra Leone OR Somalia OR South Africa OR Sudan OR Swaziland OR Tanzania OR Togo OR Uganda OR Western Sahara OR Zambia OR Zimbabwe |  |
| #4 | #1AND#2AND#3 |  |
|  | **Table 1**: Search strategy for Scopus (cont’d) |  |
| #1 | INDEXTERMS("venous thrombosis") OR INDEXTERMS("pulmonary embolism") OR INDEXTERMS("venous thromboembolism") OR INDEXTERMS("ischemic stroke") OR INDEXTERMS("myocardial infarction") OR INDEXTERMS("treatment outcome") OR INDEXTERMS(safety) OR INDEXTERMS(hemorrhage) OR INDEXTERMS(mortality) OR (  "Deep venous thrombosis" OR DVT OR "pulmonary embolism" OR PE OR "ischemic stroke" OR  "venous thromboembolism" OR VTE OR "myocardial infarction" OR MI OR "treatment outcome"   OR TTR OR INR OR "International Normalized Ratio" OR "Time in Therapeutic Range" OR "clinical effectiveness" OR efficacy OR safety OR bleeding* OR Hemorrhage* OR "major bleeding" OR "intracranial hemorrhage" OR "gastrointestinal bleeding" OR mortality OR hospital* OR  "intracranial bleeding")tiab |  |
| #2 | INDEXTERMS(Anticoagulants) OR INDEXTERMS(warfarin) OR (Anticoagulant OR "anticoagulant drugs" OR warfarin OR Coumarin OR Coumadin OR "vitamin K antagonist" OR VKA OR "oral anticoagulant" OR OAC) tiab |  |
| #3 | Africa central OR Africa eastern OR Africa southern OR Africa western OR Africa northern OR low-income country OR developing country OR middle-income country OR Sub-Sahara OR Angola OR Benin OR Botswana OR Burkina Faso OR Burundi OR Cameroon OR Cape Verde OR Central African Republic OR Chad OR Comoros OR Congo OR Brazzaville OR Cote d'Ivoire OR Djibouti OR Equatorial Guinea OR Eritrea OR Ethiopia OR Gabon OR Gambia OR Ghana OR Guinea OR Guinea-Bissau OR Kenya OR Lesotho OR Liberia OR Madagascar OR Malawi OR Mali OR Mauritania OR Mauritius OR Mozambique OR Namibia OR Niger OR Nigeria OR Rwanda OR Sao Tome and Principe OR Senegal OR Seychelles OR Sierra Leone OR Somalia OR South Africa OR Sudan OR Swaziland OR Tanzania OR Togo OR Uganda OR Western Sahara OR Zambia OR Zimbabwe |  |
| #4 | #1AND#2AND#3 |  |

Supplemental Table 2: Quality assessment

| Author | **Selection** | **Comparability** | **Outcome** |  |
| --- | --- | --- | --- | --- |
| (Anakwue et al., 2014) | ** | * | ** | 5 |
| (Mwita et al., 2018) | *** | * | ** | 6 |
| Fenta et al., 2017 | *** | * | ** | 6 |
| Yimer et al., 2021 | *** | ** | *** | 8 |
| Ahmed et al., 2017 | *** | * | *** | 7 |
| Liyew et al. - 2021 | ** | ** | *** | 7 |
| Prinsloo et al., 2021 | *** | * | ** | 6 |
| Botsile and Mwita, 2020 | *** | * | ** | 6 |
| Mariita et al., 2016 | *** | * | ** | 6 |
| Masresha et al., 2021 | *** | ** | *** | 8 |
| Sonuga et al., 2016b | **** | * | ** | 7 |
| Ouali et al., 2021 | *** | * | *** | 7 |
| Jonkman et al., 2019 | *** | * | *** | 7 |
| Ebrahim et al., 2018 | ** | * | ** | 5 |
| Getachew et al., 2023 | *** | * | *** | 7 |
