# Supplementary material for: Anticoagulation quality through time in therapeutic range in Sub-Saharan Africa: a systematic review and meta-analysis
Source: Front Med (Lausanne). 2025 Mar 14;12:1517162. doi: 10.3389/fmed.2025.1517162 (PMC11949880; doi:10.3389/fmed.2025.1517162)

Meta-regression for good TTR

library(metafor)

# Create the dataset based on the new data provided

data <- data.frame(

Authors = c("Yimer et al., 2021", "Mwita et al, 2018", "Liyew et al., 2021", "Prinsloo et al., 2021",

"Botsile and Mwita, 2020", "Masresha et al., 2021", "Ouali et al., 2021b",

"Jonkman et al., 2019a", "Ebrahim et al., 2018", "Getachew et al., 2023"),

Publication = c(2021, 2018, 2021, 2021, 2020, 2021, 2021, 2019, 2018, 2023),

Setting = c("Cardiac center", "Medical clinic", "Medical clinic", "Cardiac center",

"Cardiac center", "Medical clinic", "Cardiac center", "Medical clinic",

"Cardiac center", "Cardiac center"),

Follow_up = c(2, 2, 0.5, 1, 0.42, 2, 1, 1, 6, 0.25),

Sample_size = c(300, 410, 338, 191, 142, 202, 915, 215, 363, 347),

Total = c(300, 410, 338, 191, 142, 202, 915, 215, 363, 347),

Event = c(38, 61, 44, 34, 21, 59, 110, 22, 91, 89))

# Calculate the effect size using the Freeman-Tukey double arcsine transformation (measure = "PFT")

data <- escalc(measure = "PFT", xi = Event, ni = Total, data = data)

# Convert Setting to a binary variable for regression analysis (1 = Cardiac center, 0 = Medical clinic)

data$Cardiac_center <- ifelse(data$Setting == "Cardiac center", 1, 0)

# Perform meta-regression with Publication, Sample_size, Cardiac_center, and Follow_up as moderators

model <- rma(yi, vi, mods = ~ Publication + Sample_size + Cardiac_center + Follow_up, data = data)

# Print the summary of the model

summary(model)

Model

Mixed-Effects Model (k = 10; tau^2 estimator: REML)

logLik deviance AIC BIC AICc

6.0200 -12.0399 -0.0399 -2.3833 83.9601

tau^2 (estimated amount of residual heterogeneity): 0.0044 (SE = 0.0034)

tau (square root of estimated tau^2 value): 0.0662

I^2 (residual heterogeneity / unaccounted variability): 82.48%

H^2 (unaccounted variability / sampling variability): 5.71

R^2 (amount of heterogeneity accounted for): 31.05%

Test for Residual Heterogeneity:

QE(df = 5) = 30.3819, p-val < .0001

Test of Moderators (coefficients 2:5):

QM(df = 4) = 7.4389, p-val = 0.1144

Model Results:

estimate se zval pval ci.lb ci.ub

intrcpt -96.1205 46.1753 -2.0816 0.0374 -186.6224 -5.6186 *

Publication 0.0478 0.0229 2.0908 0.0365 0.0030 0.0926 *

Sample_size -0.0001 0.0001 -0.9972 0.3187 -0.0003 0.0001

Setting -0.0367 0.0565 -0.6492 0.5162 -0.1474 0.0741

Follow_up 0.0494 0.0205 2.4082 0.0160 0.0092 0.0897 *

---

Signif. codes: 0 ‘***’ 0.001 ‘**’ 0.01 ‘*’ 0.05 ‘.’ 0.1 ‘ ’ 1

Meta-regression for therapeutic INR

Mixed-Effects Model (k = 6; tau^2 estimator: REML)

logLik deviance AIC BIC AICc

2.0341 -4.0681 7.9319 -4.0681 91.9319

tau^2 (estimated amount of residual heterogeneity): 0 (SE = 0.0026)

tau (square root of estimated tau^2 value): 0

I^2 (residual heterogeneity / unaccounted variability): 0.00%

H^2 (unaccounted variability / sampling variability): 1.00

R^2 (amount of heterogeneity accounted for): 100.00%

Test for Residual Heterogeneity:

QE(df = 1) = 0.4003, p-val = 0.5270

Test of Moderators (coefficients 2:5):

QM(df = 4) = 34.7004, p-val < .0001

Model Results:

estimate se zval pval ci.lb ci.ub

intrcpt -11.3564 18.4400 -0.6159 0.5380 -47.4981 24.7853

Publication 0.0060 0.0091 0.6602 0.5091 -0.0119 0.0240

Sample_size -0.0007 0.0002 -3.0524 0.0023 -0.0011 -0.0002 **

Setting 0.0698 0.0481 1.4524 0.1464 -0.0244 0.1640

Follow_up -0.0212 0.0228 -0.9287 0.3531 -0.0659 0.0235

---

Signif. codes: 0 ‘***’ 0.001 ‘**’ 0.01 ‘*’ 0.05 ‘.’ 0.1 ‘ ’ 1

|  |
| --- |
| \| > \| \| --- \| |

# Sensitivity analysis for therapeutic INR

library(meta)

TherapeuticIfreeman

Myresult<-metaprop(Event,Total,data=TherapeuticIfreeman,studlab = paste(Author),method = "inverse",sm="PFT")

forest(Myresult,fixed=FALSE,random = TRUE,overall = TRUE,prediction = TRUE,pooled.events = TRUE,col.square = "red",col.square.lines = "black", xlab="Therapeutic INR", col.study = "black",col.diamond = "black")

sensitivity_analysis <- metainf(Myresult)

forest(sensitivity_analysis)


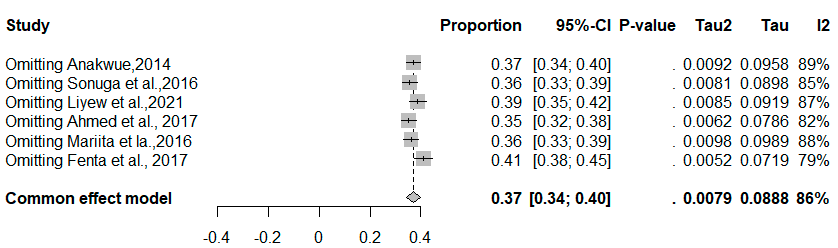


# Sensitivity analysis for good TTR


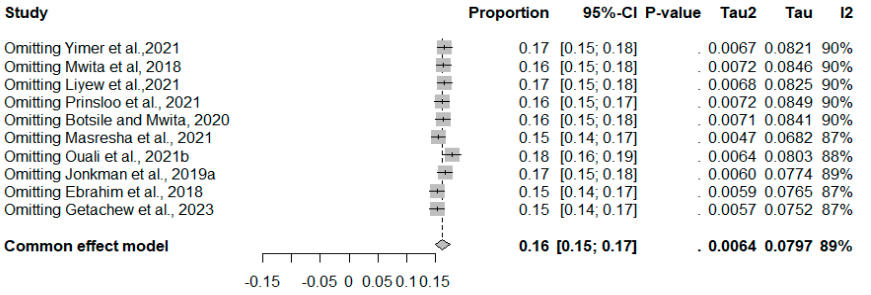

Supplement: Supplementary file 2 [file Table_2.docx]
